# MIMIC-III, a freely accessible critical care database

**DOI:** 10.1038/sdata.2016.35

**Published:** 2016-05-24

**Authors:** Alistair E.W. Johnson, Tom J. Pollard, Lu Shen, Li-wei H. Lehman, Mengling Feng, Mohammad Ghassemi, Benjamin Moody, Peter Szolovits, Leo Anthony Celi, Roger G. Mark

**Affiliations:** 1 Laboratory for Computational Physiology, MIT Institute for Medical Engineering and Science, Massachusetts Institute of Technology, Cambridge, Massachusetts 02139, USA; 2 Information Systems, Beth Israel Deaconess Medical Center, Boston, Massachusetts 02215, USA; 3 Data Analytics Department, Institute for Infocomm Research, A*STAR, Singapore 138632, Singapore; 4 Computer Science and Artificial Intelligence Laboratory, Massachusetts Institute of Technology, Cambridge, Massachusetts 02139, USA; 5 These authors contributed equally to this work

**Keywords:** Outcomes research, Health care, Prognosis, Diagnosis, Medical research

## Abstract

MIMIC-III (‘Medical Information Mart for Intensive Care’) is a large, single-center database comprising information relating to patients admitted to critical care units at a large tertiary care hospital. Data includes vital signs, medications, laboratory measurements, observations and notes charted by care providers, fluid balance, procedure codes, diagnostic codes, imaging reports, hospital length of stay, survival data, and more. The database supports applications including academic and industrial research, quality improvement initiatives, and higher education coursework.

## Background & Summary

In recent years there has been a concerted move towards the adoption of digital health record systems in hospitals. In the US, for example, the number of non-federal acute care hospitals with basic digital systems increased from 9.4 to 75.5% over the 7 year period between 2008 and 2014 (ref. 1).

Despite this advance, interoperability of digital systems remains an open issue, leading to challenges in data integration. As a result, the potential that hospital data offers in terms of understanding and improving care is yet to be fully realized. In parallel, the scientific research community is increasingly coming under criticism for the lack of reproducibility of studies^2^.

**Figure 1:**
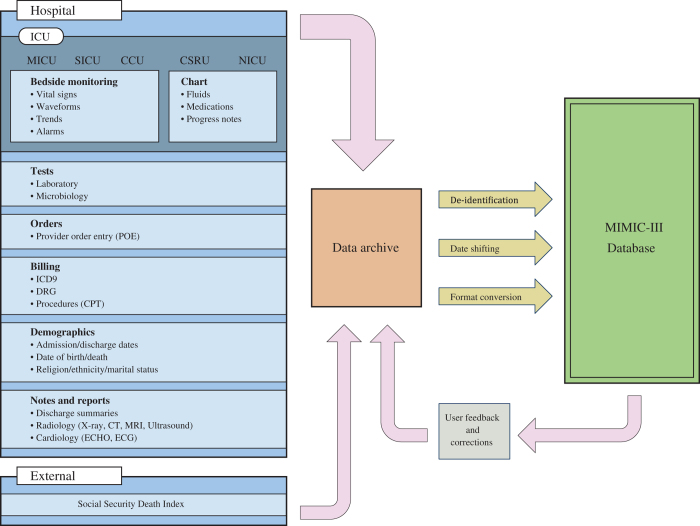
Overview of the MIMIC-III critical care database.

Here we report the release of the MIMIC-III database, an update to the widely-used MIMIC-II database (Data Citation 1). MIMIC-III integrates deidentified, comprehensive clinical data of patients admitted to the Beth Israel Deaconess Medical Center in Boston, Massachusetts, and makes it widely accessible to researchers internationally under a data use agreement (Fig. 1). The open nature of the data allows clinical studies to be reproduced and improved in ways that would not otherwise be possible.

Based on our experience with the previous major release of MIMIC (MIMIC-II, released in 2010) we anticipate MIMIC-III to be widely used internationally in areas such as academic and industrial research, quality improvement initiatives, and higher education coursework.

To recognize the increasingly broad usage of MIMIC, we have renamed the full title of the database from ‘Multiparameter Intelligent Monitoring in Intensive Care’ to ‘Medical Information Mart for Intensive Care’. The MIMIC-III critical care database is unique and notable for the following reasons:

- it is the only freely accessible critical care database of its kind;
- the dataset spans more than a decade, with detailed information about individual patient care;
- analysis is unrestricted once a data use agreement is accepted, enabling clinical research and education around the world.

### Patient characteristics

MIMIC-III contains data associated with 53,423 distinct hospital admissions for adult patients (aged 16 years or above) admitted to critical care units between 2001 and 2012. In addition, it contains data for 7870 neonates admitted between 2001 and 2008. The data covers 38,597 distinct adult patients and 49,785 hospital admissions. The median age of adult patients is 65.8 years (Q1–Q3: 52.8–77.8), 55.9% patients are male, and in-hospital mortality is 11.5%. The median length of an ICU stay is 2.1 days (Q1–Q3: 1.2–4.6) and the median length of a hospital stay is 6.9 days (Q1-Q3: 4.1–11.9). A mean of 4579 charted observations (’chartevents’) and 380 laboratory measurements (’labevents’) are available for each hospital admission. Table 1 provides a breakdown of the adult population by care unit.

The primary International Classification of Diseases (ICD-9) codes from the patient discharges are listed in Table 2. The top three codes across hospital admissions for patients aged 16 years and above were:

- 414.01 (‘Coronary atherosclerosis of native coronary artery’), accounting for 7.1% of all hospital admissions;
- 038.9 (‘Unspecified septicemia’), accounting for 4.2% of all hospital admissions; and
- 410.71 (‘Subendocardial infarction, initial episode of care’), accounting for 3.6% of all hospital admissions.

### Classes of data

**Figure 2:**
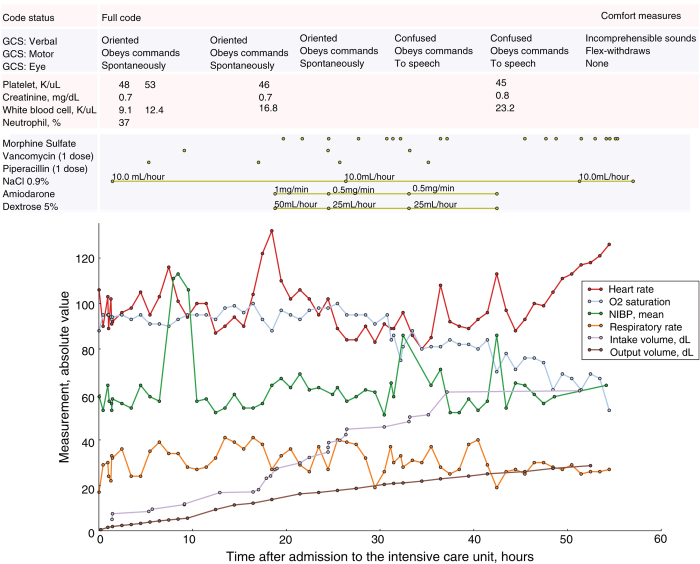
Sample data for a single patient stay in a medical intensive care unit. GCS is Glasgow Coma Scale; NIBP is non-invasive blood pressure; and O2 saturation is blood oxygen saturation.

Data available in the MIMIC-III database ranges from time-stamped, nurse-verified physiological measurements made at the bedside to free-text interpretations of imaging studies provided by the radiology department. Table 3 gives an overview of the different classes of data available. Figure 2 shows sample data for a single patient stay in a medical intensive care unit. The patient, who was undergoing a course of chemotherapy at the time of admission, presented with febrile neutropenia, anemia, and thrombocytopenia.

## Methods

The Laboratory for Computational Physiology at Massachusetts Institute of Technology is an interdisciplinary team of data scientists and practicing physicians. MIMIC-III is the third iteration of the MIMIC critical care database, enabling us to draw upon prior experience with regard to data management and integration^3^.

### Database development

The MIMIC-III database was populated with data that had been acquired during routine hospital care, so there was no associated burden on caregivers and no interference with their workflow. Data was downloaded from several sources, including:

- archives from critical care information systems.
- hospital electronic health record databases.
- Social Security Administration Death Master File.

Two different critical care information systems were in place over the data collection period: Philips CareVue Clinical Information System (models M2331A and M1215A; Philips Health-care, Andover, MA) and iMDsoft MetaVision ICU (iMDsoft, Needham, MA). These systems were the source of clinical data such as:

- time-stamped nurse-verified physiological measurements (for example, hourly documentation of heart rate, arterial blood pressure, or respiratory rate);
- documented progress notes by care providers;
- continuous intravenous drip medications and fluid balances.

With exception to data relating to fluid intake, which differed significantly in structure between the CareVue and MetaVision systems, data was merged when building the database tables. Data which could not be merged is given a suffix to denote the data source. For example, inputs for patients monitored with the CareVue system are stored in INPUTEVENTS_CV, whereas inputs for patients monitored with the Metavision system are stored in INPUTEVENTS_MV. Additional information was collected from hospital and laboratory health record systems, including:

- patient demographics and in-hospital mortality.
- laboratory test results (for example, hematology, chemistry, and microbiology results).
- discharge summaries and reports of electrocardiogram and imaging studies.
- billing-related information such as International Classification of Disease, 9th Edition (ICD-9) codes, Diagnosis Related Group (DRG) codes, and Current Procedural Terminology (CPT) codes.

Out-of-hospital mortality dates were obtained using the Social Security Administration Death Master File. A more detailed description of the data is shown in Table 1. Physiological waveforms obtained from bedside monitors (such as electrocardiograms, blood pressure waveforms, photoplethysmograms, impedance pneumograms) were obtained for a subset of patients.

Several projects are ongoing to map concepts within the MIMIC database to standardized dictionaries. For example, researchers at the National Library of Medicine National Institutes of Health have mapped laboratory tests and medications in MIMIC-II to LOINC and RxNorm, respectively^4^. Efforts are also underway to transform MIMIC to common data models, such as the Observational Medical Outcomes Partnership Common Data Model, to support the application of standardized tools and methods^5^. These developments are progressively incorporated into the MIMIC database where possible.

The project was approved by the Institutional Review Boards of Beth Israel Deaconess Medical Center (Boston, MA) and the Massachusetts Institute of Technology (Cambridge, MA). Requirement for individual patient consent was waived because the project did not impact clinical care and all protected health information was deidentified.

### Deidentification

Before data was incorporated into the MIMIC-III database, it was first deidentified in accordance with Health Insurance Portability and Accountability Act (HIPAA) standards using structured data cleansing and date shifting. The deidentification process for structured data required the removal of all eighteen of the identifying data elements listed in HIPAA, including fields such as patient name, telephone number, address, and dates. In particular, dates were shifted into the future by a random offset for each individual patient in a consistent manner to preserve intervals, resulting in stays which occur sometime between the years 2100 and 2200. Time of day, day of the week, and approximate seasonality were conserved during date shifting. Dates of birth for patients aged over 89 were shifted to obscure their true age and comply with HIPAA regulations: these patients appear in the database with ages of over 300 years.

Protected health information was removed from free text fields, such as diagnostic reports and physician notes, using a rigorously evaluated deidentification system based on extensive dictionary look-ups and pattern-matching with regular expressions^6^. The components of this deidentification system are continually expanded as new data is acquired.

### Code availability

The code that underpins the MIMIC-III website and documentation is openly available and contributions from the research community are encouraged: https://github.com/MIT-LCP/mimic-website

A Jupyter Notebook containing the code used to generate the tables and descriptive statistics included in this paper is available at: https://github.com/MIT-LCP/mimic-iii-paper/

## Data Records

MIMIC-III is a relational database consisting of 26 tables (Data Citation 1). Tables are linked by identifiers which usually have the suffix ‘ID’. For example, SUBJECT_ID refers to a unique patient, HADM_ID refers to a unique admission to the hospital, and ICUSTAY_ID refers to a unique admission to an intensive care unit.

Charted events such as notes, laboratory tests, and fluid balance are stored in a series of ‘events’ tables. For example the OUTPUTEVENTS table contains all measurements related to output for a given patient, while the LABEVENTS table contains laboratory test results for a patient.

Tables prefixed with ‘D_’ are dictionary tables and provide definitions for identifiers. For example, every row of CHARTEVENTS is associated with a single ITEMID which represents the concept measured, but it does not contain the actual name of the measurement. By joining CHARTEVENTS and D_ITEMS on ITEMID, it is possible to identify the concept represented by a given ITEMID. Further detail is provided below.

### Data tables

Developing the MIMIC data model involved balancing simplicity of interpretation against closeness to ground truth. As such, the model is a reflection of underlying data sources, modified over iterations of the MIMIC database in response to user feedback. Table 4 describes how data is distributed across the data tables. Care has been taken to avoid making assumptions about the underlying data when carrying out transformations, so MIMIC-III closely represents the raw hospital data.

Broadly speaking, five tables are used to define and track patient stays: ADMISSIONS; PATIENTS; ICUSTAYS; SERVICES; and TRANSFERS. Another five tables are dictionaries for cross-referencing codes against their respective definitions: D_CPT; D_ICD_DIAGNOSES; D_ICD_PROCEDURES; D_ITEMS; and D_LABITEMS. The remaining tables contain data associated with patient care, such as physiological measurements, caregiver observations, and billing information.

In some cases it would be possible to merge tables—for example, the D_ICD_PROCEDURES and CPTEVENTS tables both contain detail relating to procedures and could be combined—but our approach is to keep the tables independent for clarity, since the data sources are significantly different. Rather than combining the tables within MIMIC data model, we suggest researchers develop database views and transforms as appropriate.

## Technical Validation

The number of structural changes were minimized to achieve the desired level of deidentification and data schema, helping to ensure that MIMIC-III closely represents the raw data collected within the Beth Israel Deaconess Medical Center.

Best practice for scientific computing was followed where possible^7^. Code used to build MIMIC-III was version controlled and developed collaboratively within the laboratory. This approach encouraged and facilitated sharing of readable code and documentation, as well as frequent feedback from colleagues.

Issue tracking is used to ensure that limitations of the data and code are clearly documented and are dealt with as appropriate. The research community is encouraged to report and address issues as they are found, and a system for releasing minor database updates is in place.

## Usage Notes

### Data access

MIMIC-III is provided as a collection of comma separated value (CSV) files, along with scripts to help with importing the data into database systems including PostreSQL, MySQL, and MonetDB. As the database contains detailed information regarding the clinical care of patients, it must be treated with appropriate care and respect. Researchers are required to formally request access via a process documented on the MIMIC website^8^. There are two key steps that must be completed before access is granted:

- the researcher must complete a recognized course in protecting human research participants that includes Health Insurance Portability and Accountability Act (HIPAA) requirements.
- the researcher must sign a data use agreement, which outlines appropriate data usage and security standards, and forbids efforts to identify individual patients.

Approval requires at least a week. Once an application has been approved the researcher will receive emails containing instructions for downloading the database from PhysioNetWorks, a restricted access component of PhysioNet^9^.

### Example usage

MIMIC has been used as a basis for coursework in numerous educational institutions, for example in medical analytics courses at Stanford University (course BIOMEDIN215), Massachusetts Institute of Technology (courses HST953 and HST950J/6.872), Georgia Institute of Technology (course CSE8803), University of Texas at Austin (course EE381V), and Columbia University (course G4002), amongst others. MIMIC has also provided the data that underpins a broad range of research studies, which have explored topics such as machine learning approaches for prediction of patient outcomes, clinical implications of blood pressure monitoring techniques, and semantic analysis of unstructured patient notes^10–13^.

A series of 'datathons' have been held alongside development of the MIMIC database. These events assemble caregivers, data scientists, and those with domain-specific knowledge with the aim of creating ideas and producing clinically relevant, reproducible research^14^. In parallel the events introduce new researchers to MIMIC and provide a platform for continuous review and development of code and research.

Documentation for the MIMIC database is available online^8^. The content is under continuous development and includes a list of studies that have been carried out using MIMIC. The website includes functionality that enables the research community to directly submit updates and improvements via GitHub.

### Collaborative research

Our experience is that many researchers work independently to produce code for data processing and analysis. We seek to move towards a more collaborative, iterative, and self-checking development process where researchers work together on a shared code base. To facilitate collaboration, a public code repository has been created to encourage researchers to develop and share code collectively: https://github.com/MIT-LCP/mimic-code.

The repository has been seeded with code to calculate commonly utilized variables in critical care research, including severity of illness scores, comorbidity scores, and duration of various treatments such as mechanical ventilation and vasopressor use. We encourage users to incorporate this code into their research, provide improvements, and add new contributions that have potential to benefit the research community as a whole. Over time, we expect the repository to become increasingly vital for researchers working with the MIMIC-III database.

Alongside work on the centralized codebase, we support efforts to transform MIMIC into common data models such the Observational Medical Outcomes Partnership Common Data Model (OMOP-CDM)^5^. Developing these common models may help to facilitate integration with complementary datasets and to enable the application of generalized analytic tools. Important efforts to map concepts to standardized clinical ontologies are also underway.

## Additional Information

**How to cite this article:** Johnson, A. E. W. *et al.* MIMIC-III, a freely accessible critical care database. *Sci. Data* 3:160035 doi: 10.1038/sdata.2016.35 (2016).

## Supplementary Material



## Figures and Tables

**Table 1:** Details of the MIMIC-III patient population by first critical care unit on hospital admission for patients aged 16 years and above.

**Critical care unit**	**CCU**	**CSRU**	**MICU**	**SICU**	**TSICU**	**Total**
Distinct patients, no. (% of total admissions)	5,674 (14.7%)	8,091 (20.9%)	13,649 (35.4%)	6,372 (16.5%)	4,811 (12.5%)	38,597 (100%)
Hospital admissions, no. (% of total admissions)	7,258 (14.6%)	9,156 (18.4%)	19,770 (39.7%)	8,110 (16.3%)	5,491 (11.0%)	49,785 (100%)
Distinct ICU stays, no. (% of total admissions)	7,726 (14.5%)	9,854 (18.4%)	21,087 (39.5%)	8,891 (16.6%)	5,865 (11.0%)	53,423 (100%)
Age, years, median (Q1-Q3)	70.1 (58.4–80.5)	67.6 (57.6–76.7)	64.9 (51.7–78.2)	63.6 (51.4–76.5)	59.9 (42.9–75.7)	65.8 (52.8–77.8)
Gender, male, % of unit stays	4,203 (57.9%)	6,000 (65.5%)	10,193 (51.6%)	4,251 (52.4%)	3,336 (60.7%)	27,983 (55.9%)
ICU length of stay, median days (Q1-Q3)	2.2 (1.2–4.1)	2.2 (1.2–4.0)	2.1 (1.2–4.1)	2.3 (1.3–4.9)	2.1 (1.2–4.6)	2.1 (1.2–4.6)
Hospital length of stay, median days (Q1-Q3)	5.8 (3.1–10.0)	7.4 (5.2–11.4)	6.4 (3.7–11.7)	7.9 (4.4–14.2)	7.4 (4.1–13.6)	6.9 (4.1–11.9)
ICU mortality, percent of unit stays	685 (8.9%)	353 (3.6%)	2,222 (10.5%)	813 (9.1%)	492 (8.4%)	4,565 (8.5%)
Hospital mortality, percent of unit stays	817 (11.3%)	424 (4.6%)	2,859 (14.5%)	1,020 (12.6%)	628 (11.4%)	5,748 (11.5%)
CCU is Coronary Care Unit; CSRU is Cardiac Surgery Recovery Unit; MICU is Medical Intensive Care Unit; SICU is Surgical Intensive Care Unit; TSICU is Trauma Surgical Intensive Care Unit.						

**Table 2:** Distribution of primary International Classification of Diseases, 9th Edition (ICD-9) codes by care unit for patients aged 16 years and above.

**Critical care unit**	**CCU stays, No. (% by unit)**	**CSRU stays, No. (% by unit)**	**MICU stays, No. (% by unit)**	**SICU stays, No. (% by unit)**	**TSICU stays, No. (% by unit)**	**Total stays, No. (% by unit)**
Infectious and parasitic diseases, i.e., septicemia, other infectious and parasitic diseases, etc., (001–139)	305 (4.2%)	72 (0.8%)	3,229 (16.7%)	448 (5.6%)	152 (2.8%)	4,206 (8.6%)
Neoplasms of digestive organs and intrathoracic organs, etc., (140–239)	126 (1.8%)	287 (3.2%)	1,415 (7.3%)	1,225 (15.3%)	466 (8.6%)	3,519 (7.2%)
Endocrine, nutritional, metabolic, and immunity (240–279)	104 (1.4%)	36 (0.4%)	985 (5.1%)	178 (2.2%)	54 (1.0%)	1,357 (2.8%)
Diseases of the circulatory system, i.e., ischemic heart diseases, diseases of pulmonary circulation, dysrhythmias, heart failure, cerebrovascular diseases, etc., (390–459)	5,131 (71.4%)	7,138 (78.6%)	2,638 (13.6%)	2,356 (29.5%)	684 (12.6%)	17,947 (36.6%)
Pulmonary diseases, i.e., pneumonia and influenza, chronic obstructive pulmonary disease, etc., (460–519)	416 (5.8%)	141 (1.6%)	3,393 (17.5%)	390 (4.9%)	225 (4.1%)	4,565 (9.3%)
Diseases of the digestive system (520–579)	264 (3.7%)	157 (1.7%)	3,046 (15.7%)	1,193 (14.9%)	440 (8.1%)	5,100 (10.4%)
Diseases of the genitourinary system, i.e., nephritis, nephrotic syndrome, nephrosis, and other diseases of the genitourinary system (580–629)	130 (1.8%)	14 (0.2%)	738 (3.8%)	101 (1.3%)	31 (0.6%)	1,014 (2.1%)
Trauma (800–959)	97 (1.3%)	494 (5.4%)	480 (2.5%)	836 (10.5%)	2,809 (51.7%)	4,716 (9.6%)
Poisoning by drugs and biological substances (960–979)	50 (0.7%)	2 (0.0%)	584 (3.0%)	58 (0.7%)	11 (0.2%)	705 (1.4%)
Other	565 (7.9%)	739 (8.1%)	2,883 (14.9%)	1,204 (15.1%)	563 (10.4%)	5,954 (12.1%)
Total	7,188 (14.6%)	9,080 (18.5%)	19,391 (39.5%)	7,989 (16.3%)	5,435 (11.1%)	49,083 (100%)
CCU is Coronary Care Unit; CSRU is Cardiac Surgery Recovery Unit; MICU is Medical Intensive Care Unit; SICU is Surgical Intensive Care Unit; TSICU is Trauma Surgical Intensive Care Unit.						

**Table 3:** Classes of data available in the MIMIC-III critical care database.

**Class of data**	**Description**
Billing	Coded data recorded primarily for billing and administrative purposes. Includes Current Procedural Terminology (CPT) codes, Diagnosis-Related Group (DRG) codes, and International Classification of Diseases (ICD) codes.
Descriptive	Demographic detail, admission and discharge times, and dates of death.
Dictionary	Look-up tables for cross referencing concept identifiers (for example, International Classification of Diseases (ICD) codes) with associated labels.
Interventions	Procedures such as dialysis, imaging studies, and placement of lines.
Laboratory	Blood chemistry, hematology, urine analysis, and microbiology test results.
Medications	Administration records of intravenous medications and medication orders.
Notes	Free text notes such as provider progress notes and hospital discharge summaries.
Physiologic	Nurse-verified vital signs, approximately hourly (e.g., heart rate, blood pressure, respiratory rate).
Reports	Free text reports of electrocardiogram and imaging studies.

**Table 4:** An overview of the data tables comprising the MIMIC-III (v1.3) critical care database.

**Table name**	**Description**
ADMISSIONS	Every unique hospitalization for each patient in the database (defines HADM_ID).
CALLOUT	Information regarding when a patient was cleared for ICU discharge and when the patient was actually discharged.
CAREGIVERS	Every caregiver who has recorded data in the database (defines CGID).
CHARTEVENTS	All charted observations for patients.
CPTEVENTS	Procedures recorded as Current Procedural Terminology (CPT) codes.
D_CPT	High level dictionary of Current Procedural Terminology (CPT) codes.
D_ICD_DIAGNOSES	Dictionary of International Statistical Classification of Diseases and Related Health Problems (ICD-9) codes relating to diagnoses.
D_ICD_PROCEDURES	Dictionary of International Statistical Classification of Diseases and Related Health Problems (ICD-9) codes relating to procedures.
D_ITEMS	Dictionary of local codes (’ITEMIDs’) appearing in the MIMIC database, except those that relate to laboratory tests.
D_LABITEMS	Dictionary of local codes (’ITEMIDs’) appearing in the MIMIC database that relate to laboratory tests.
DATETIMEEVENTS	All recorded observations which are dates, for example time of dialysis or insertion of lines.
DIAGNOSES_ICD	Hospital assigned diagnoses, coded using the International Statistical Classification of Diseases and Related Health Problems (ICD) system.
DRGCODES	Diagnosis Related Groups (DRG), which are used by the hospital for billing purposes.
ICUSTAYS	Every unique ICU stay in the database (defines ICUSTAY_ID).
INPUTEVENTS_CV	Intake for patients monitored using the Philips CareVue system while in the ICU, e.g., intravenous medications, enteral feeding, etc.
INPUTEVENTS_MV	Intake for patients monitored using the iMDSoft MetaVision system while in the ICU, e.g., intravenous medications, enteral feeding, etc.
OUTPUTEVENTS	Output information for patients while in the ICU.
LABEVENTS	Laboratory measurements for patients both within the hospital and in outpatient clinics.
MICROBIOLOGYEVENTS	Microbiology culture results and antibiotic sensitivities from the hospital database.
NOTEEVENTS	Deidentified notes, including nursing and physician notes, ECG reports, radiology reports, and discharge summaries.
PATIENTS	Every unique patient in the database (defines SUBJECT_ID).
PRESCRIPTIONS	Medications ordered for a given patient.
PROCEDUREEVENTS_MV	Patient procedures for the subset of patients who were monitored in the ICU using the iMDSoft MetaVision system.
PROCEDURES_ICD	Patient procedures, coded using the International Statistical Classification of Diseases and Related Health Problems (ICD) system.
SERVICES	The clinical service under which a patient is registered.
TRANSFERS	Patient movement from bed to bed within the hospital, including ICU admission and discharge.

## References

[d1] The MIMIC-III Clinical Database Pollard T. J. Johnson A. E. W. 2016 http://dx.doi.org/10.13026/C2XW26

